# Acute effect of different doses of caffeinated chewing gum on exercise performance in caffeine-habituated male soccer players

**DOI:** 10.3389/fnut.2023.1251740

**Published:** 2023-10-18

**Authors:** Ulas Can Yildirim, Neslihan Akcay, Dan Iulian Alexe, Ozcan Esen, Mehmet Gulu, Cristina Cîrtiţă-Buzoianu, Fahri Safa Cinarli, Marilena Cojocaru, Cengizhan Sari, Cristina Ioana Alexe, Raci Karayigit

**Affiliations:** ^1^Faculty of Sport Sciences, Sinop University, Sinop, Türkiye; ^2^Hasan Doğan School of Physical Education and Sports, Karabük University, Karabük, Türkiye; ^3^Department of Physical and Occupational Therapy, Faculty of Movement, Sports and Health, Sciences, Vasile Alecsandri University of Bacau, Bacău, Romania; ^4^Department of Sport, Exercise and Rehabilitation, Northumbria University, Newcastle upon Tyne, United Kingdom; ^5^Faculty of Sport Sciences, Kırıkkale University, Kırıkkale, Türkiye; ^6^Faculty of Letters, Communication and Public Relations Department, Vasile Alecsandri University of Bacau, Bacău, Romania; ^7^Faculty of Sport Sciences, İnönü University, Malatya, Türkiye; ^8^Faculty of Physical Education and Sport, Spiru Haret University of Bucuresti, Bucharest, Romania; ^9^Faculty of Sport Sciences, Mus Alparslan University, Mus, Türkiye; ^10^Department of Physical Education and Sports Performance, Faculty of Movement, Sports and Health, Sciences, Vasile Alecsandri University of Bacau, Bacau, Romania; ^11^Faculty of Sport Sciences, Ankara University, Ankara, Türkiye

**Keywords:** team sports, strength, vertical jump, ball kicking speed, gum, ergogenic aid

## Abstract

The ergogenic benefits of caffeine have been well established, but there is scarce research on its chewing gum form. The present research aimed to examine the effects of different doses (100 and 200 mg) of caffeinated chewing gum on muscle strength, vertical jump performance, and ball-kicking speed in trained male soccer players. In a double-blind, randomized counterbalanced, and crossover research design, 14 male soccer players (age = 22 ± 2 y; body mass = 74.2 ± 7.1 kg; height = 180.0 ± 6.8 cm; habitual caffeine intake = 358.9 ± 292.4 mg/day) participated in three experimental trials. In each trial, participants performed isometric handgrip strength, quadriceps and hamstring strength, ball-kicking speed, and 15 s countermovement jump test 10 min after chewing 100 mg (LCAF) or 200 mg (MCAF) of caffeinated gum or placebo (PLA). MCAF improved quadriceps strength (53.77 ± 5.77 kg) compared to LCAF (49.62 ± 8.81 kg, *p* = 0.048) and PLA (49.20 ± 7.20 kg, *p* = 0.032). However, neither LCAF nor MCAF had a significant effect on the isometric handgrip and hamstring strength, ball-kicking speed, and 15 s countermovement jump test (all *p* > 0.05). These findings support chewing gum as an alternative mode of caffeine administration which can be used as a nutritional ergogenic aid for trained soccer players, at least for quadriceps strength.

## 1. Introduction

Caffeine is a widely utilized ergogenic aid in the athletic population, and, over the past several decades, a substantial body of research has been dedicated to exploring the physiological, metabolic, and performance-related impacts of caffeine (1). The literature has extensively established that caffeine intake can enhance athletic performance across multiple sports, with recommended dosages ranging from 3 to 6 mg/kg (2–5). Nonetheless, the administration of this supplementation regimen may not be universally applicable to all athletes, given the time-related constraints and potential for serious side effects (6). Moreover, caffeine ingestion remains a trending topic in the field, as researchers still investigate whether various moderating factors, such as the efficacy of lower doses (≤ 3 mg/kg), level of caffeine habituation, and forms of caffeine (e.g., coffee, chewing gum or anhydrous form), can modify the immediate ergogenic benefits of caffeine (7, 8).

In most of the research, caffeine has been administered in either capsule or tablet forms ~60 min before an exercise protocol to allow for full absorption and reaching peak plasma caffeine concentrations (8). Chewing gum has been most recently used as another delivery method as it can provide faster absorption and, therefore, results in a shorter duration for potential responses compared to more traditional modes of ingestion (9). Unlike capsule form, caffeinated chewing gum is absorbed directly into the bloodstream via the buccal cavity, bypassing first-pass metabolism, which results in significant absorption of caffeine in only 5–10 min (10). This faster absorption would minimize the risk of gastrointestinal disorders (6) and genotypic disadvantages in athletes (CYP1A2, CC allele carriers) (11). In this regard, several previous studies (12, 13) reported that administration of caffeine gum 5 to 15 min before exercise improved aerobic endurance (4, 14), cycling time trials (15), repeated sprints (16), jumping and strength (5) and team-sport specific battery tests (17). Additionally, a meta-analysis by Barreto et al. (18) showed that caffeinated chewing gum can be beneficial for trained athletes involving endurance or strength/power type exercises; however, none of the 13 studies included in that meta-analysis explored the dose–response relationship of caffeine gum and exercise performance. Evaluating dose–responses of caffeine gum on exercise performance is important to provide accurate practical recommendations.

Although several studies examined the effect of caffeine supplementation on the physical performance of soccer players (19), according to the best of our knowledge, only one study investigated the impact of caffeinated chewing gum on male soccer players (17). While Ranchordas et al. (3) reported that caffeinated (200 mg) gum enhanced Yo-Yo intermittent recovery test level 1 and countermovement jump (CMJ) performance, the effect of caffeinated gum on maximum strength remains to be determined. Furthermore, kicking speed, which is another key skill and performance variable in soccer (20, 21), was examined only by one study in which an anhydrous form of caffeine (3 mg/kg) ingestion had no effect in futsal players (22). The fact that ~11% of a soccer match is composed of sprints (10–15 meters every 90 s) (23) and ~ 80% of the last movements of the soccer players before the goal are high-intensity movements (24) show the importance of muscle power in soccer. A number of studies investigating the relationship between power and high-intensity performance report a positive correlation between the two parameters (25, 26). Caffeine’s ability to improve muscle strength by increasing neuromuscular activity makes it an attractive ergogenic aid for soccer players. Furthermore, given that 97% of English professional soccer players consume caffeine prior to training or matches (27) and considering the practicality of this form of administration, caffeine supplementation via chewing gum form would be a beneficial nutritional strategy for soccer players. Therefore, more research is required to confirm or/and extend the findings from previous research and to draw a more solid conclusion if and to what extent the chewing gum mode of caffeine supplementation would be effective for soccer players.

Emerging evidence suggests that a lower caffeine dose (< 3 mg/kg) could provide similar ergogenic benefits on exercise performance but with fewer side effects such as headache, gastrointestinal discomfort, tachycardia, or insomnia (6), which would be advantageous for athletes. However, it is also suggested that a high level (> 231 ± 88 mg/day) of habitual caffeine consumption can be a factor that may induce tolerance to the acute benefits of caffeine (28), and therefore athletes need to consume pre-exercise doses greater than that habitually consumed (7). While the existing literature on the effect of habituation on the ergogenicity of caffeine is conflicting, it is apparent that data is needed on whether caffeinated chewing gum would provide an ergogenic effect in caffeine-habituated individuals. To address these notable gaps in the literature, the present research aimed to examine the effects of different doses (100 and 200 mg) of caffeinated chewing gum on muscle strength, vertical jump performance, and ball-kicking speed in trained male soccer players. It was hypothesized that 200 mg, but not 100 mg, of caffeinated gum would elicit important benefits on exercise performance.

## 2. Methods

### 2.1. Participants

The study included 14 highly trained male soccer players (mean ± SD, age = 22 ± 2 y; body mass = 74.2 ± 7.1 kg; height = 180.0 ± 6.8 cm; body fat percentage = 11.4 ± 3.9%; habitual caffeine intake = 358.9 ± 292.4 mg/day) with 11 ± 1 years of soccer training experience. The sample size was calculated utilizing the statistical program G*Power (version 3.1.9.4; Dusseldorf, Germany) with the following variables: ANOVA, repeated-measures, within-factors, effect size f (ES) for 0.24, alpha = 0.05, power (1-error probability) = 0.90, statistical power = 90%, *r* = 0.85, and one set of participants. Three test sessions were used in the statistical test. According to the power analysis, a sample size of at least 13 people was required to detect statistically significant differences in strength, jumping, and ball-kicking performance between 100 or 200 mg of caffeinated chewing gum and placebo conditions. Soccer players were competing in the interprovince league (semi-professional) and had at least 4 days of training sessions and one match per week. Participants did not currently, or in the previous 3 months, have a musculoskeletal injury, and did not use any ergogenic aid (i.e., creatine, beta-alanine, and nitrate) that might have affected the muscular responses in the previous 3 months. Since goalkeepers’ movement patterns throughout gameplay are unique, they were excluded. A validated questionnaire developed by Bühler et al. (29) was adapted to quantify habitual caffeine consumption, and participants were asked to report the frequency with which they consumed various caffeine-containing meals and supplements. All participants were fully informed about the risks of the study and gave written informed consent. The study was approved by the Mus Alparslan University Ethic Committee (approval no: 95187) in accordance with the latest version of the Declaration on Helsinki.

### 2.2. Pre-experimental standardizations

Participants recorded their 24-h food intake before the first trial. The records were then photocopied and participants were provided with copies to follow the same diet for the subsequent sessions. Participants were also asked to refrain from alcohol consumption and severe physical activity 24 h before each trial. Prior to each session, verbal confirmation was obtained regarding compliance with the prescribed diet and avoidance protocols. To simulate real-life sport-setting conditions (27), participants were advised to maintain their standard daily caffeine consumption routines throughout the study to avoid “abstinence” effects (7).

### 2.3. Experimental design

In a randomized, double-blind, counterbalanced, and crossover research design, participants attended one familiarization and three test sessions that included caffeinated chewing gum or placebo gum 15 min before testing. The process of randomization and blinding was conducted by an independent researcher who did not participate in the data collection. In each of the experimental conditions, the participants were given (I) gum containing 100 mg of caffeine (one stick of caffeinated and one stick of non-caffeinated chewing gum, LCAF); (II) gum containing 200 mg of caffeine (two sticks of caffeinated gum, MCAF); or (III) placebo (two sticks of non-caffeinated chewing gum, PLA). A standardized dose of 100 or 200 mg of caffeine was administered to all participants using one or two sticks of commercially available caffeinated chewing gum (Military Energy Gum; MarketRight Inc., Plano, IL, United States). The chewing gum was given directly into the mouths of the participants from an opaque container to mitigate the potential influence of visual cues stemming from the appearance of each gum. The chewing gums, whether caffeinated or non-caffeinated, were split into small cubes measuring approximately 0.3 cm in length to hide their physical characteristics. Placebo was a readily accessible, non-caffeinated gum with a similar flavor, color, and shape (Mentos, Turkey). Test sessions were separated by 48–72 h to allow recovery. For all visits, participants presented to the laboratory in the morning hours (08,00–09,00) after 10 h of night fasting. Participants were required to continuously chew the gum for 5 mins and then expel the gum into a container. A similar caffeinated gum supplementation protocol was previously performed by Ryan et al. (13). A standardized warm-up was performed consisting of 5 min of jogging and 5 min of dynamic stretching (heel flicks, high knees, leg swings, walking lunges, and marching). Afterward, handgrip and quadriceps, hamstring maximum strength, ball-kicking speed, and 15-s CMJ tests were conducted. Immediately after the completion of the experimental trials, participants were asked if they experienced any side effects. Furthermore, at the end of three experimental sessions, participants were also requested to guess the trial “order.”

### 2.4. Isometric handgrip and quadriceps-hamstring strength

A calibrated handgrip dynamometer (Takei 5,101, Tokyo, Japan) was utilized to assess maximum handgrip strength. The participants were instructed to assume a standing position and grasp the handgrip digital dynamometer while exerting their maximum isometric force with their dominant hand (determined as the preferred arm to throw a ball with) and with their elbow and wrist extended at the side of their body. Subsequently, the individuals were directed to execute a flexion of their fingers and maintain the flexed position by engaging in a maximal isometric contraction lasting for 5 s. The experimental protocol involved the execution of two trials, each interspersed by a 60-s rest period. The highest value was used as maximal force. Participants were strongly urged to exert their maximum strength (30, 31).

Quadriceps and hamstring strength were measured at 90° of knee flexion using a handheld dynamometer (Nicholas Manual Muscle Tester, Lafayette Instrument Co, Lafayette, Ind.), which has been previously compared against isokinetic dynamometers and demonstrated to be a valid measure of muscular strength (32). The method employed for measuring strength involves determining the maximum force that a participant can withstand at a 90° angle, quantified in kilograms. For quadriceps strength, participants were asked to sit on a high table with their arms crossed on their chest, feet free, and knees flexed to 90°. For hamstring strength, participants were asked to lie face down on a flat table with their knees flexed to 90° and arms free. Each measurement was replicated two times (~60 s apart) and the same researcher performed all the measurements. The dynamometer was placed at 2 cm proximal of the ankle on the leg and the highest values obtained were recorded as maximum quadriceps and hamstring force. The handheld dynamometer has demonstrated remarkable interday and intraday reliability (33).

### 2.5. Ball-kicking speed

The speed of the ball kick was assessed at a distance of 11 meters from the goal using radar gun equipment (Stalker Solo 2, model ATS, Plano, Texas, United States). The device has the capability to measure speeds within the range of 16–177 km/h and has a sensitivity of ±2 km/h. At the outset, the participants’ dominant legs were subjectively determined and recorded to ensure maximal speed. Subsequently, the individuals executed the act of shooting the ball utilizing the instep kick method. Shooting involved the utilization of a ball that adhered to FIFA regulations, specifically a size 5 soccer ball that is recommended for individuals aged 12 years and above. The ball-kicking speed was measured by the researcher who consistently operated the radar gun across all trials to ensure test reliability. The measurements were taken from behind the goal from a central spot similar to the penalty spot from where the soccer player executed the kick. The participants were instructed to strive for precision in their kicks, aiming to hit the designated target, while simultaneously achieving the highest possible velocity in their ball-kicking movements. Two attempts (~60 s apart) were allotted to each participant to attain the optimal score, and the outcomes were documented in km/h.

### 2.6. 15 s countermovement jump test

The CMJ test was conducted for 15 s and utilized a mobile contact mat (Smart Jump; Fusion Sport, Queensland, Australia). Only one attempt was allowed in the test. Participants initiated the experiment in a vertical stance, with their body mass equally distributed across both lower extremities and their upper limbs situated at the level of their waist. Following an auditory cue, the individuals assumed a squatting position whereby their knees were flexed at a 90° angle. They then executed a vertical jump with maximal effort and landed bilaterally, repeating this sequence of jumping for a duration of 15 s. The participants were given the directive to perform a vertical jump with maximal effort for a duration of 15 s. Peak and mean jump height (cm) and peak and mean power (watt/kg) were recorded (34).

### 2.7. Statistical analysis

The data analysis was conducted using IBM SPSS 22.0 software (IBM Corp., Armonk, New York, United States) and presented as means ± SD. The normality of the distribution was verified through the Shapiro–Wilk test. A one-way analysis of variance (ANOVA) with repeated measures was used to assess differences in performance variables following caffeinated gums and PLA. The partial eta square (η_p_^2^) was utilized to assess the effect size, which was classified as small (0.10–0.24), moderate (0.25–0.39), or large (≥0.40). The sphericity assumption was subjected to analysis using Mauchly’s test, followed by the application of the Greenhouse–Geisser adjustment in cases where it was deemed necessary. If there was a significant main or interaction effect, Bonferroni corrected paired *t-*tests were applied as *post hoc* paired comparisons. Cohen’s *d* effect sizes were computed for every paired comparison, with effect sizes ranging from very small (0.20–0.49), moderate (0.50–0.79) to large (≥0.80) (35). In order to evaluate the consistency of the three test sessions, intraclass correlation coefficients (ICC) were computed and subsequently interpreted according to established guidelines; poor reliability: >0.5, moderate reliability: 0.5–0.75, good reliability: 0.75–0.90, and excellent reliability: >0.90 (36).

## 3. Results

**Figure 1:**
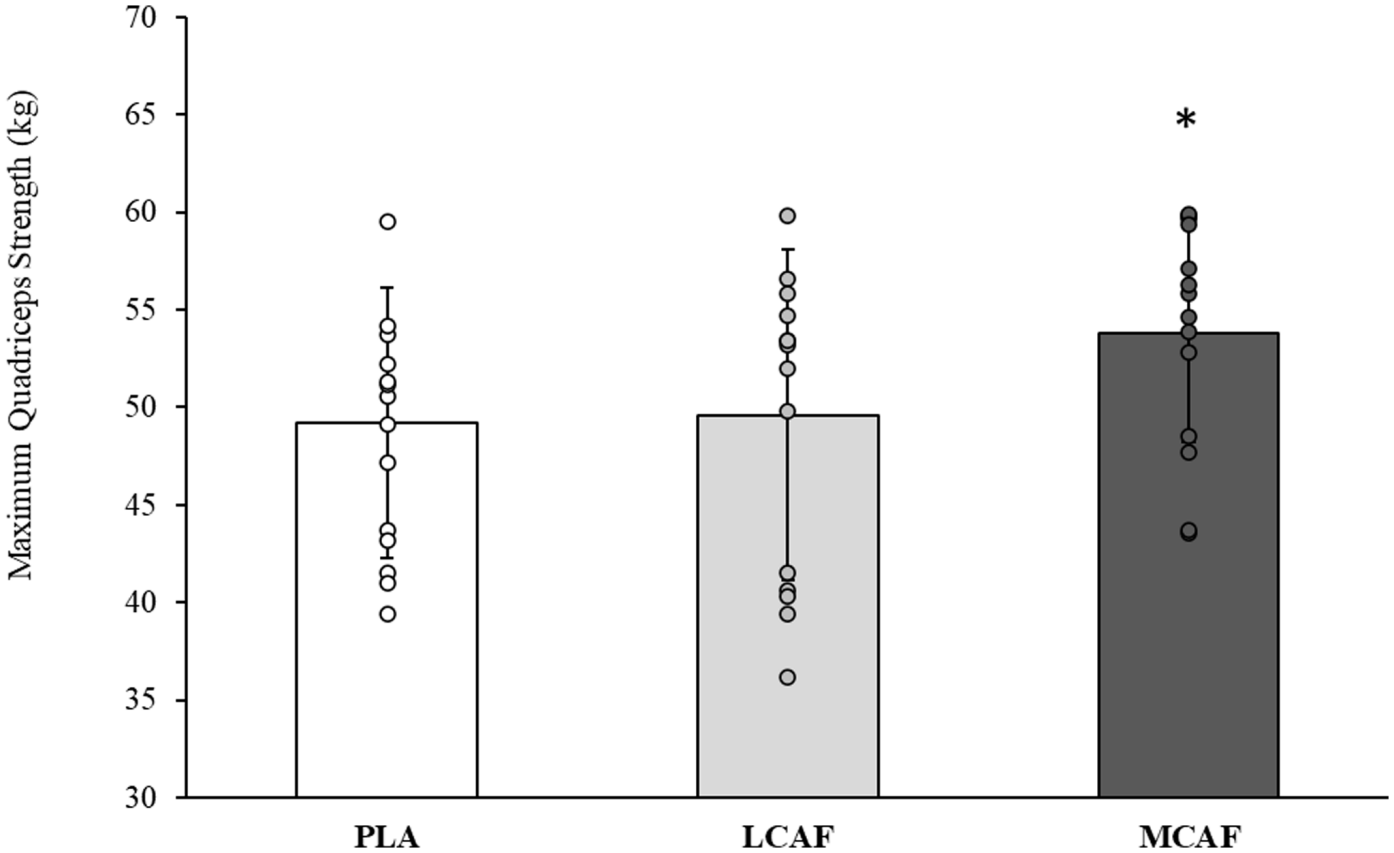
Isometric quadriceps strength performance during the PLA, LCAF, and MCAF. PLA, placebo; LCAF, 100 mg of caffeinated gum; MCAF, 200 mg of caffeinated gum. *, significantly different from PLA and LCAF.

There was a significant difference in quadriceps strength performance between conditions (*p* = 0.025; η_p_^2^ = 0.248). Bonferroni *post-hoc* analysis showed quadriceps strength was greater in MCAF (53.77 ± 5.77 kg) than PLA (49.20 ± 7.20 kg, *p* = 0.032; 95% CI = 0.35–8.78; *d* = 0.70) and LCAF trials (49.62 ± 8.81 kg, *p* = 0.048; 95% CI = 0.03–8.27; *d* = 0.55). ICC values were found to be “good” (0.82). There was no difference in hamstring strength between conditions (*p* = 0.251; η_p_^2^ = 0.101). ICC values were detected as “good” (0.74). One-way ANOVA showed no significant differences in the isometric handgrip strength (*p* = 0.145; η_p_^2^ = 0.138; Table 1). Despite non-significance, mean values of LCAF (48.16 kg, Δ = 2.31%; *d* = 0.16) and MCAF (48.76 kg, Δ = 3.59%; *d* = 0.25) were superior to PLA (47.07 kg) which may have a practical meaning for the field settings. ICC values were also found to be “excellent” (0.95). Ball-kicking speed was also not significantly different between conditions (*p* = 0.658; η_p_^2^ = 0.032; Table 1). ICC values showed “good” reliability (0.76). Peak (*p* = 0.678; η_p_^2^ = 0.029) and mean (*p* = 0.833; η_p_^2^ = 0.014) jump heights were also not different between conditions. ICC values showed “moderate” reliability for peak (0.57) and mean (0.65) jump heights. Furthermore, peak (*p* = 0.337; η_p_^2^ = 0.080) and mean (*p* = 0.439; η_p_^2^ = 0.061) power during the 15-s CMJ test was similar between conditions (Table 1). ICC values were also detected to be “good” (0.76) to “moderate” (0.72), respectively (Figure 1).

**Table 1:** Handgrip and hamstring strength, ball kicking, and countermovement jump performance outcomes in placebo (PLA), low caffeinated (LCAF) gum, and medium caffeinated (MCAF) gum trials.

	PLA	LCAF	MCAF
Ball-kicking (km/h)	106.74 ± 7.69	107.80 ± 4.34	106.36 ± 5.79
Handgrip strength (kg)	47.07 ± 6.82	48.16 ± 6.22	48.76 ± 6.53
Hamstring strength (kg)	25.66 ± 3.49	28.11 ± 6.12	26.81 ± 5.83
Peak jump height (cm)	37.27 ± 4.57	37.48 ± 7.39	34.62 ± 5.42
Mean jump height (cm)	32.43 ± 3.95	32.23 ± 5.55	30.20 ± 6.31
Peak power (Watt/Kg)	45.91 ± 6.26	46.12 ± 7.85	43.70 ± 6.00
Mean power (Watt/Kg)	41.86 ± 5.01	41.69 ± 5.74	40.04 ± 6.05

## 4. Discussion

To the best of our knowledge, this is the first time that the effect of different doses of caffeine (100 vs. 200 mg), in the form of chewing gum, is investigated on isometric muscle strength, vertical jump performance, and ball-kicking speed in male soccer players. The main finding of the current study was that 200 mg (~2.6 mg/kg) of caffeinated chewing gum improved quadriceps strength “*moderately*” by 8.3% (*d* = 0.55) and 9.2% (*d* = 0.70) compared to 100 mg of caffeinated chewing gum and placebo, respectively. However, no effect of caffeine was observed on hamstring and handgrip strength, vertical jump performance, and ball-kicking speed.

The current results showed that 200 mg of caffeine gum administered 10 min before exercise is an effective method of caffeine delivery to enhance quadriceps strength in caffeine-habituated male soccer players. As this study represents one of the earliest investigations into the impact of caffeinated chewing gum on strength performance, the ability to draw comparisons between our findings and those of identical studies is presently constrained. To the best of the authors’ knowledge, there is only one previous investigation that has been conducted with a similar design. Venier et al. (5) administrated 300 mg caffeine gum to 19 resistance-trained men 10 min prior to test sessions and observed an enhancement in peak torque in the knee extensors by 3.6% (*d* = 0.21). The present investigation, in conjunction with the research by Venier et al. (5), supports the ergogenic benefit of caffeine gum on knee extensors. However, it is necessary to conduct further research on the impact of caffeinated chewing gum on muscular strength performance with different exercise modes (e.g., isokinetic and dynamic) to draw a more solid conclusion about the effects of this form of caffeine on muscular strength. These results are in line with a previous meta-analysis (37) and an experimental study (37) that demonstrated the beneficial impact of caffeine administered in capsule form on the knee extensor muscles.

On the other hand, the same benefit of 200 mg caffeine gum was not detected for hamstring strength in the present study. The knee extensors exhibit a lower level of neural activation, ranging from 85 to 95%, during maximal voluntary contractions (38). This creates an opportunity for caffeine to facilitate greater muscle recruitment centrally, which is not observed in other muscle groups that already exhibit activation levels of 90 to 95%, leaving minimal to no room for improvement (37). The findings of the current study would be attributed to the central influence theory, which suggests that caffeine supplementation enhances strength by stimulating the central nervous system (22). This is due to the greater increase in motor unit recruitment and firing rates observed in larger muscle groups compared to smaller ones, which may be attributed to the varying number of adenosine receptors present in skeletal muscles size (39). Furthermore, although it is not a soccer-specific test, isometric handgrip strength is a widely used, well-known test for detecting changes in maximal strength production. Caffeinated gum showed no statistically significant improvement in handgrip strength in the current investigation. However, it is important to emphasize that the percentage of increase (3.59%) and the effect size (*d* = 0.25) in the 200 mg of caffeinated chewing gum condition were comparable to the vast majority of previous research (with anhydrous caffeine) in this topic (30, 31, 39). Recently, a meta-analysis by Grgic (40) concluded that 1–3 mg/kg of anhydrous caffeine is ergogenic for isometric handgrip strength with a small effect size (*d* = 0.20). The present research revealed no improvement in ball-kicking speed after chewing either 100 or 200 mg of caffeine gum.

In the context of soccer match play, the execution of individual vertical jumping bouts is a crucial aspect (41). However, players are subjected to prolonged intermittent activity, as they are obligated to perform repeated vertical jumping (42). The current study showed that there were no improvements in 15 s of CMJ performance following either 100 or 200 mg of caffeinated chewing gum. Two meta-analyses that addressed the relationship between caffeine ingestion and vertical jump performance have recently been published (43, 44). While our result is in line with one of these two meta-analyses, which found the acute intake of caffeine does not affect vertical jump performance (43), it is opposed to the other meta-analysis, which reported that even minimal doses of caffeine (1 and 2 mg/kg) can have an ergogenic effect on jumping height (37). These differences in results between studies may be due to the variance of some methodological conditions, such as participants included, the methodological quality of the included studies, and habitual caffeine consumption. Nevertheless, it is noteworthy that in those meta-analyses, the studies that were incorporated administered caffeine to the subjects through either liquid or capsule forms. To our knowledge, there are only a limited number of studies that have investigated the effects of chewing gum on jumping performance. These studies have produced varying and contradictory results, with some indicating potential benefits (5, 17, 45, 46) and others showing no significant effects (3). In order to compare directly with previous studies, in the present study, the vertical jump test was performed with the hands on the hips and without arm swings, which may have moderated our results. Nevertheless, available data (45) suggests that the inclusion of arm swing during the CMJ would result in an increase in jump height among volleyball players after 6.4 mg/kg of caffeine administration via chewing gum. Furthermore, the absence of enhancement in CMJ performance in the present study could plausibly be linked to a few moderating factors, including athletic/competition level, caffeine consumption rate of the subjects, and the caffeine doses used (1). In addition, a meta-analysis by Barreto et al. (18), which investigated the effects of caffeine chewing gum on exercise performance, suggested that ≥3 mg/kg of caffeine is necessary to elicit performance gains. However, the present study was designed to evaluate the effects of “very low” doses [100 mg (1.34 mg/kg) and 200 mg (2.68 mg/kg)] of caffeine via chewing gum, which seems to moderate our results.

Although no previous research has analyzed the effect of caffeinated chewing gum on the ball-kicking speed in soccer players, our finding is in agreement with a previous study that administered 3 mg/kg of anhydrous caffeine in futsal players (22). Similar findings were also reported in other similar sports-specific situations such as in basketball free throws (47), volleyball service (45), and specific tasks or simulated matches in ball games (48). In light of the results of the current and previous research, it appears reasonable to propose that the pre-exercise or pre-match chewing of caffeine gum does not yield any discernible impact on enhancing ball velocity during shooting among male soccer players, although further research is warranted to draw a firm conclusion. The high technical component that influences ball speed in kicking may have a moderating effect on the benefits of caffeine chewing gum. Therefore, coaches should consider the use of caffeine for improving upper limb strength levels.

The current investigation, despite its strengths, exhibits several limitations. First, the inability to measure electromyography (EMG) or voluntary activation (%) during quadriceps and hamstring strength measurements makes it difficult to fully elucidate the exact mechanisms of 200 mg of caffeinated chewing gum on quadriceps strength. Second, in order to ensure consistency in calorie consumption prior to testing sessions, a 24-h dietary record was employed and replicated. While this method may be defined as acceptable, it is worth noting that macronutrient intake preceding each trial was not subjected to software analysis. Third, data is required regarding direct measures of performance such as sprints or changes of direction, which were not measured in the current research due to constraints in laboratory conditions. Lastly, rather than relative to body weight, absolute amounts of caffeine (100 and 200 mg) were given due to the technical limitations, which resulted in subjects ingesting a caffeine dose between 1.20 to 1.66 mg/kg for 100 mg condition and between 2.40 to 3.33 mg/kg for 200 mg condition. The variability in relative dosage and likely different caffeine absorption from the gum (49) may have led to the inter-individual heterogeneity in responses.

## 5. Conclusion

In conclusion, ingestion of 200 mg caffeine, in the form of caffeinated gum, 10 min before the test improved maximum quadriceps strength but did not benefit hamstring and handgrip strength, CMJ, and ball-kicking speed in caffeine-habituated trained male soccer players. These findings support chewing gum as an alternative mode of caffeine administration, which can be used as a nutritional ergogenic aid for trained soccer players, at least for quadriceps strength.

## Data availability statement

The raw data supporting the conclusions of this article will be made available by the authors, without undue reservation.

## Ethics statement

The studies involving humans were approved by the Mus Alparslan University Ethic committee (approval no: 95187). The studies were conducted in accordance with the local legislation and institutional requirements. The participants provided their written informed consent to participate in this study.

## Author contributions

UY, RK, NA, DA, and CA: conceptualization. FC, MG, OE, CS, CC-B, and MC: methodology. OE, RK, UY, DA, and FC: software. UY, RK, NA, MG, MC, and CA: validation. NA, OE, MG, FC, RK, and CS: formal analysis. RK, UY, NA, and FC: investigation. UY and NA: resources. UY, RK, OE, and MG: data curation. RK, UY, OE, MG, CA, CC-B, and MC: writing—original draft preparation. DA, MC, CC-B, CA, DA, and OE: writing—review and editing. RK, OE, UY, and CS: visualization. DA, MC, CA, CC-B, and DA: supervision. OE, UY, RK, and DA: project administration. DA and MC: funding acquisition. All authors contributed to the article and approved the submitted version.
